# Social theory and science diplomacy

**DOI:** 10.12688/openreseurope.15020.1

**Published:** 2024-01-08

**Authors:** Rasmus Gjedssø BERTELSEN

**Affiliations:** 1 Department of Social Sciences, UiT The Arctic University of Norway, Tromsø, Troms og Finnmark, 9037, Norway

**Keywords:** Social theory, International Relations theory, power, science diplomacy, history, strategy

## Abstract

This essay discusses the relationship between social theory and science diplomacy for both academic and policy application. This discussion is based on workpackage 2 Power with Science Diplomacy of H2020 Inventing a shared Science Diplomacy for Europe (InsSciDE) and consortium-wide discussions.

The outcome of the discussions on theory of science diplomacy is that it is unfeasible to develop one theory of science diplomacy. Science diplomacy practice is rich and wide-ranging. Science diplomacy as a concept continues to be contested and there is no consensus on a definition, which makes for dynamic research and debate. The conceptual instability of science diplomacy complicates defining it. After defining science diplomacy, it remains unclear what about science diplomacy to theorize.

Ideal types of science diplomacy practices address the definitional challenge for the time being and allow theorizing which brings order to rich empirical material and links science diplomacy practices to diplomacy analytically rather than normatively. Looking at science diplomacy as an independent, intermediary, or dependent variable contributes to theorizing it.

## Introduction

This deliverable concludes the work of Work Package 2 Power with Science Diplomacy of the H2020 Inventing a shared Science Diplomacy for Europe. This deliverable builds on the theory discussions and development of WP2 and points the road forward for theory considerations for science diplomacy. The composition of the InsSciDE consortium ensured stimulating debate.

The immediate conclusion is that it is not feasible to develop one theory of science diplomacy. The practice of science diplomacy is rich and wide-ranging, and discussions continue to focus on defining science diplomacy. The concept of science diplomacy continues to be contested, which contributes to the dynamism of research and discussions. It is difficult to formulate a singular theory of an unstable object. Beyond defining science diplomacy, theorizing science diplomacy requires deciding what to theorize.

This paper outlines independent-dependent variable relations between science diplomacy practices and diplomatic goals, which indicates avenues for theorizing science diplomacy. The paper also connects science diplomacy with social theory and International Relations theory on power, which contributes concepts and theory for understanding science diplomacy outcomes. Political psychology for science diplomacy is also introduced for understanding science diplomacy. The relationship between science diplomacy and world order is discussed. Finally, future theorizing of science diplomacy is introduced.

Theory is a conceptual framework to understand the relationship between factors, actors, and conditions. Theory identifies assumptions about relationships between variables, which actors are more or less important, and conditions for validity. Theory can be seen as a map. A map does not show every detail, for then it would be unreadable; it shows the most important features. Theories should be parsimonious, explain a few important things, and identify a few important actors.

Theory of science diplomacy should therefore take as a starting point looking at what is most important, what is to be explained concerning science diplomacy. What relations between what variables should be explained? What outcomes should be explained? Who are the relevant actors and the necessary conditions for these outcomes?

Science diplomacy is a rich area of practice as also illustrated by H2020 InsSciDE case studies ( Mays *et al.*, 2022) and broader scholarship. It is uncertain if it is currently possible and useful to seek to formulate one theory to explain this rich practice of science diplomacy ( Mays, 2022).

Based on discussions led in InsSciDE and with other sources during the period of Horizon 2020 projects on science diplomacy, this deliverable instead proposes to explore the interfaces between social theory and science diplomacy practice for a mutual deeper understanding. Social theory contributes to understanding science diplomacy practice, and science diplomacy practice contributes to understanding social theory and in particular International Relations theory.

## A definition of science diplomacy for theorizing

Science diplomacy is a contested concept with a rich debate that seeks to define it. It is useful to briefly delve into the two constituent terms, “science” and “diplomacy”. The main term is “diplomacy”, which is the pursuit of state foreign policy interest through diplomatic actions. Below I refer to the Vienna Convention on Diplomatic Relations to define diplomatic functions. Diplomacy generally has a positive connotation, especially compared to war, but it is important to keep in mind how both diplomacy and war are instruments of statecraft to pursue state foreign policy interest.

“Science” is an adjective to “diplomacy”, so science diplomacy is a subgroup of diplomatic action. I will use the D2.6 European Science Diplomacy Strategy definition by Dr Björn Fägersten: science diplomacy is the use of science and science cooperation for foreign policy purposes ( Fägersten, 2021).

“Science” means different things in different languages. “Science” in English generally connotes natural or physical sciences and technology and related disciplines, and often fails to include social science, humanities, law, business, theology, and similar disciplines. In contrast, in, for example, German and Scandinavian languages, “Wissenschaft”, or “videnskab/vitenskap/vetenskap” (literally meaning creation of knowledge) cover humanities, natural or social sciences, technology, health, etc. In principle and practice, all academic disciplines are used in science diplomacy as pursuit of state foreign policy interest, not least social sciences, and humanities, so the broader definition is followed here.

“Science” in general, and of particular interest here for science diplomacy, is also a contested concept. Within and across societies there is an idealized view of science as neutral and objective. The idealized view of science is a basic – often implicit – assumption behind views that science can provide a neutral and objective language to solve international political conflicts. This in turn reflects an assumption that international political conflicts are caused by misunderstanding and miscommunication because of a less neutral political or diplomatic language. Such an interpretation fails to understand the anarchic nature of international politics. There is no world government to protect states against each other, like the state domestically ideally enforces the law and protects individuals and entities. States are forced into self-help and jealously watching other states because of this anarchic nature of the international system, which is not a communication problem.

In contrast, Science and Technology Studies alongside other scholarship has critically shown the politicized nature of science, how science is a product of power and politics, and how science contributes to power and politics ( Jasanoff, 2004). While the idealized view of science explains many expressions around science diplomacy, the critical view of science is necessary to understand the nature of science diplomacy.

## Science diplomacy, the state, and non-state actors

The state is a key actor since science diplomacy is the use of science for foreign policy purposes. However, it is also clear that science diplomacy is a practice involving countless non-state actors. The relationship between the state and non-state actors in science diplomacy therefore becomes a central question for theorizing science diplomacy.

Historically, states have been and continue to be (relatively) very powerful actors, while there have been and continue to be very powerful non-state actors. One militarily very powerful non-state actor was the British East India Company of the 1700s and 1800s. States and non-state actors cooperate, compete, and co-opt each other at times.

The visible role of non-state actors in science diplomacy can give the impression of independence of these actors vis-à-vis the state. The apparent independence of non-state actors in science diplomacy can be related to the normative, optimistic, or even naïve view of science diplomacy. In times of deep crisis, the real power relationship between the state and non-state actors in science diplomacy can become clear and often shows the relative power of the state. Such expressions of state power and interference in science cooperation and exchange stand in contrast to views of science diplomacy of independence of the state and de-escalation and bridge-building between opposing states.

A historical example of state power vis-à-vis non-state actors is when the US Government and the People’s Republic of China during the Korean War dismantled the 20+ American missionary higher education institutions in Mainland China that had functioned as independent bi-cultural academic institutions. These US missionary higher education institutions had been academically successful and built powerful bridges between American and Chinese society. Neither the US Government nor the People’s Republic of China accepted such bi-cultural institutions in their zero-sum struggle between liberal democracy and global capitalism and communism and decolonization. These institutions were cut off from US philanthropic funding by US sanctions on Mainland China, and the People’s Republic of China did not tolerate such independent academic institutions; they were nationalized ( Fairbank, 1974; West, 1976; Xu, 2009). Today, Western governments have generally instituted academic boycotts of Russia in response to the Russian invasion of Ukraine. These boycotts seem to be motivated by intolerance of collaborating with Russian institutions and not by expectation of effect on the battlefield in Ukraine or on Russian leadership preferences.

Science diplomacy practice shows how science diplomacy by non-state actors can be used as a tool by the state, materially backed by the state, and politically tolerated by the state. French support for the Jesuit *Université Saint-Joseph de Beyrouth* since 1883, and US support for the Protestant missionary American University of Beirut and the American University in Cairo since World War II, are illustrative examples of great power material support for non-state actors for regional foreign policy reasons ( Bertelsen, 2012; Bertelsen, 2014a; Bertelsen, 2014b; Eddé, 2000; ). Today, cross-border science and higher education cooperation are mainly funded from public resources.

Non-state actors with significant material and intellectual resources can engage in science diplomacy seemingly independent of the state. Large charities are such examples, where American foundations such as the Rockefeller, Ford, Carnegie, etc., Foundations have been significant science diplomacy actors in US foreign relations. As pointed out by Inderjeet Parmar, these foundations have close relations to the US foreign policy establishment and point to questions on the relations of the state and especially economically powerful actors ( Parmar, 2012). Weak states and materially poorer societies have much fewer resources and possibilities, also in science diplomacy, making a Biblical point that strong states with rich societies have all, and weak states with poor societies lose all.

An example of a materially, intellectually, and politically resourceful de facto non-state actor is the Catholic church in general and particularly the Jesuit Order, which has exercised global science diplomacy in coexistence with Western and Eastern states for centuries. Faith-based organizations and missionary activities have been important science diplomacy non-state actors, which is clear from countless higher education institutions around the world with missionary roots.

## Theorizing science diplomacy as statecraft

Science diplomacy as foreign policy by means of science and scientific cooperation is a relationship that lends itself to theorizing. Independent and dependent variables can be formulated, and relationships between them evaluated. Looking at science diplomacy as an independent, intermediary, or dependent variable sheds light on science diplomacy.

Science diplomacy practice as an independent variable and foreign policy outcomes as a dependent variable is one important way to look at and theorize science diplomacy. Considering the continued contested nature of science diplomacy, it is also relevant to look at science diplomacy as the dependent variable to be explained.

History and current observation of science diplomacy show the breadth and depth of this practice. To provide a rough overview of science diplomacy practice, I will refer to the 2010 Royal Society/American Association for the Advancement of Science (RS/AAAS) typology of science diplomacy ( The Royal Society & AAAS, 2010). This typology is much debated and criticized in science diplomacy discussion. However, it is well-known and useful to capture science diplomacy practices. This typology distinguishes between three ideal types of science diplomacy practices (which are also to some extent science diplomacy goals). These three ideal types are useful, because they are distinct and non-overlapping. There may well be additional ideal types of science diplomacy practices to be formulated, but it is necessary that any additional ideal type categories of science diplomacy practices be distinct and not overlap with any other categories. The criticism of the RS/AAAS typology is partly based on overlooking the ideal type nature of these categories and a lack of distinction between practices and goals of science diplomacy.

RS/AAAS’ first ideal type science diplomacy practice is “science IN diplomacy,” which is applying or inserting scientific expertise in diplomacy, typically negotiations. “Science IN diplomacy” can also be thought of as a normative goal of science-based and informed negotiation and decision-making. In the first instance, science diplomacy as science in diplomacy is an independent variable to explain a foreign policy outcome. In the second instance, science in diplomacy as a normative goal of science-based, informed negotiations and decision-making is the dependent variable to explain. The second view implicitly reflects the above-mentioned idealized view of science and the assumptions that objective, neutral science can contribute to resolution of conflicts caused by miscommunication and misunderstanding.

RS/AAAS’ second ideal type science diplomacy practice is “diplomacy FOR science,” where states conduct diplomacy to facilitate science cooperation. According to Fägersten, “diplomacy FOR science” is relevant for looking at science diplomacy to the extent that it is an intermediary variable explaining “science IN diplomacy” or the third ideal type practice “science FOR diplomacy” ( Fägersten, 2021).

RS/AAAS’ third ideal type science diplomacy practice of “science FOR diplomacy” gets much attention in research and policy discussions on science diplomacy. “Science FOR diplomacy” corresponds to the transnational epistemic communities between societies created through scientific activities ( Haas, 1992). These transnational relations can be deep, personal, and at high academic and societal levels. In my own research, I have pointed to American and French universities in the Middle East and East Asia as “information and resource bridges” between the Middle Eastern and East Asian host societies and American and French society in academia, government, civil society, and business ( Bertelsen, 2014b).

These “science FOR diplomacy” relations are often seen in contrast to political conflict between states. These relations are seen as de-escalatory, resilient, and independent of state-level political conflict. Here, the above-mentioned state-power for science and higher education relations between societies should be kept in mind.

There are continuous historical examples where both democratic and autocratic states have limited intellectual relations with an opposing society. Today, China and Russia, for domestic political reasons, significantly limit intellectual ties with the West. Liberal democracies in the West have for some time been reconsidering intellectual ties especially with China in much more restrictive ways ( Danish Security and Intelligence Service (PET), 2021). Currently, Western governments are generally obliging their scientific communities to cut intellectual ties with Russia because of the Russian invasion of Ukraine.

As mentioned, there is much debate and criticism of the RS/AAAS definition of science diplomacy. Alternative definitions of science diplomacy do not add clarity in my view. For instance, Gluckman *et al.* attempt to define science diplomacy based on general foreign policy goals of national needs, cross-border issues, and global needs and challenges ( Gluckman *et al.*, 2017), without specifying science diplomacy practice compared to other diplomatic practice. To grasp science diplomacy practice, we must keep in mind how the main noun is “diplomacy” and “science” is the adjective, so science diplomacy is a sub-sum of diplomacy. What is diplomacy in general and what is science diplomacy in particular?

Diplomacy as general practice is defined by article 3 of the 1961 Vienna Convention on Diplomatic Relations as: ”1. The functions of a diplomatic mission consist, inter alia, in:

(a) Representing the sending State in the receiving State; (b) Protecting in the receiving State the interests of the sending State and of its nationals, within the limits permitted by international law; (c) Negotiating with the Government of the receiving State; (d) Ascertaining by all lawful means conditions and developments in the receiving State, and reporting thereon to the Government of the sending State; (e) Promoting friendly relations between the sending State and the receiving State, and developing their economic, cultural and scientific relations.”

A simple matrix and keeping in mind both state and non-state actors shows that the three main practices of science diplomacy to varying degrees form part of all five diplomatic functions according to the Vienna Convention ( Table 1). Such a matrix represents ideal types, and empirical science diplomacy is more difficult to place in categories, but ideal types are useful for ordering and understanding the social world. An infinitely detailed map becomes unreadable.

**Table 1.** Matrix for the five diplomatic functions.

	Representation	Protection	Negotiation	Information	Friendship
Science IN diplomacy	Negotiating is a key diplomatic function to employ “science IN diplomacy.” Scientific expertise can be employed on a continuum from competitive manner in a zero-sum game to a cooperative manner in a positive-sum game to address a common problem, such as climate change.				
Diplomacy FOR science	States protect and promote their tangible and intangible scientific interests through “Diplomacy FOR science.” This science diplomacy practice is the state diplomacy practice in science diplomacy, so it is to varying extent part of presentation, protection, negotiation, information-gathering, and building friendly relations.				
Science FOR diplomacy	Transnational epistemic communities in “science FOR diplomacy” can be particularly useful in information-gathering and promoting general friendly relations. However, scientists do not usually represent their state, protect state interests, nor formally negotiate for their state, which would bring these scientists outside “science FOR diplomacy.”				

## Social theory linking science diplomacy and general diplomacy

General social theory on power and International Relations theory more narrowly can serve to elucidate the nexus between science diplomacy and general diplomacy. InsSciDE has contributed to develop conceptual and theoretical understanding of this nexus through continuous exchanges and discussions throughout the project.

Under conditions of competitive international politics, where the survival of the state is ultimately at stake, power is a key concept. Power is also a highly contested concept for social analysis, which is the root of a century-long discussion developing this concept ( Baldwin, 2002). First of all, it is important to keep in mind that power is a relational concept, it is about the relationship between two actors. In this vein, it is important to focus on outcomes, A achieving desired behavior with B. It is tempting to see power as a resource question, where the relative resources of A and B simply can be compared, but outcomes often may not reflect resources, which are therefore misleading.

The starting point of the social theory debate on power is “direct power” (later identified as the 1 ^st^ face of power), where A can force B to do what B would not otherwise do ( Dahl, 1961; Weber, 1921-1922). Direct power overlaps with what Joseph Nye in his sophisticated discussions of power for a broader academic and policy audience terms “hard power”, military coercion or economic inducement (which is in opposition to “soft power” to be discussed below) ( Nye, 2011). Nye also distinguishes between zero-sum power over an opponent and power with an opponent (to solve common problems). Direct or hard power is often in the background of diplomacy with implicit or explicit threats or inducements of violence, sanctions, or inducement.

Direct, hard power may seem less relevant concerning science diplomacy. It should be kept in mind that science can be very costly, especially space, nuclear, ocean, polar, and similar science is the prerogative of superpowers or great powers or major corporations. Wealthier states and actors make decisions that impose themselves on less wealthy states and societies. Luk van Langenhove has pointed out that “science IN diplomacy” can be thought of as competitive use of science and technology in a zero-sum game against diplomatic opponents ( Langenhove, 2017). “Science IN diplomacy” does not necessarily need to be cooperative and positive-sum games of solving common global challenges such as climate change, where the Intergovernmental Panel on Climate Change can be seen as “science IN diplomacy.”

The social theory debate on power moved significantly forward with Peter Bachrach and Morton S. Baratz seminal 1962 and 1963 papers, where they introduced the 2 ^nd^ face of power or agenda-setting power ( Bachrach & Baratz, 1962; Bachrach & Baratz, 1963). It may not be a case that a more powerful A forces B to do as it wants, but A keeps B’s interests off the agenda (which B is aware of). As social theory of power moves from direct, hard power to more subtle forms of power, it becomes more relevant for science diplomacy and starts to engage with Science and Technology Studies’ critical examination of science (in contrast to idealized views of science).

Agenda-setting power or “non-decision power” is interesting for linking science diplomacy practices and diplomacy. “Science IN diplomacy” offers opportunities for shaping agendas, including, or excluding topics according to actors’ interests. “Diplomacy FOR science” will also entail agenda-setting (power). Transnational epistemic communities in “science FOR diplomacy” will have both agenda-setting questions among their members, but also have agenda-setting power towards outsiders. Epistemic communities are noteworthy for their agenda-setting power on a policy question ( Adler & Haas, 1992). International organizations and international civil society may be seen as avenues for less resourceful states and actors to promote their interests and agendas. This argument must be examined critically as more resourceful states and actors may have even better possibilities to shape agendas and institutions.

Steven Lukes advanced the social theory discussion of power with the 3 ^rd^ face of power, conscience-controlling power, where A shapes B’s perceptions of power, without B realizing so ( Lukes, 1974). Lukes’ insights on power points to Michel Foucault’s work on knowledge and power, how power shapes what is considered knowledge/truth, and vice-versa knowledge is a source of power ( Foucault, 1975; Foucault, 1980). These considerations also relate to Pierre Bourdieu’s work on social, cultural and financial capital as well as symbolic violence to protect the upper (middle) class’ position through the educational system at the expense of working class ( Bourdieu, 1979; Bourdieu, 1989). The critical insights of Science and Technology Studies highlight the power and politics in science. All in all, these theoretical insights shed light on science diplomacy and diplomacy.

Science holds power to define what is true and good, so superior science and technology resources hold the “science IN diplomacy” potential to shape what is acceptable and true. This power is also present in “diplomacy FOR science” shaping true and acceptable agendas. The socialization potential of members of transnational epistemic “science FOR diplomacy” communities is also an example of this 3 ^rd^ face of power or power-knowledge nexus.

Education holds the possibility to shape others’ views and perceptions and to promote and exclude different groups. The 1st, 2nd and 3rd faces of power are in play here. A more powerful party can make decisions on the educational opportunities of less powerful parties (1st face). The more powerful party can overtly shape the agenda and content of this education (2nd face) and less overtly socialize the less powerful party through such education (3rd face). These mechanisms have characterized colonial and proselytizing education.

In the 1990s and early 2000s, Nye’s concept of “soft power” gained significant academic and policy attention, although much attention was misunderstood and focused on soft power resources rather than behavioral outcomes ( Nye, 2004). Nye’s soft power is power through attraction as opposed to hard power based on coercion or inducement. Post 9/11 and in the context of the War on Terror, soft power attracted much policy interest as the non-violent alternative to counter fundamentalist Islamism ( Lord, 2006; Rugh, 2006). Real soft power, changing B’s behavior or views based on attraction, is probably more limited than expected, especially in the optimistic post-9/11 policy literature on soft power. The American and French universities in the Middle East show that Middle Easterners understand well the detrimental effects of US and French Middle East policy on their communities and region, but high-quality education with improved life conditions is acknowledged and sought after ( Bertelsen, 2012; Eddé, 2000).

Nye has written successfully on power for a broader academic and policy audience than the academic debate outlined here ( Nye, 2011). Nye distinguishes between power over (zero-sum games) and power with (positive-sum games), where power with is the ability to solve common problems with a counterpart rather than coerce or manipulate the counterpart (1 ^st^, 2 ^nd^, 3 ^rd^ face of power). With power over, the resources of the counterpart may be a problem; with power with, the lack of resources of the counterpart may rather be the problem. Many chaotic transnational challenges such as climate change, pandemics, migration, and crime, are to be addressed with counterparts rather than forcing or manipulating them. For instance, the European Union is faced with poorer and unstable states to its south and east (except now Russia), which the EU has addressed through Neighborhood Policies to build capacity in these countries.

Positive-sum games of power with a counterpart are relevant for understanding science diplomacy as a diplomatic tool. Global North states with more resources have used and continue to use science diplomacy as a means of solving common problems with Global South states through all three RS/AAAS practices of science diplomacy. “Science IN diplomacy” can be central in such capacity-building neighborhood policies, which is often the topic of “diplomacy FOR science.” This policy by funding cooperation between Global North and Global South states builds transnational epistemic communities in “science FOR diplomacy.”

Other concepts of power have emerged beyond and besides the classical debate on the three faces of power. One such concept is the (sometimes unintended) structural power from deciding concerning structures ( Guzzini, 1993). Science in its broad *Wissenschaft* sense is a global, complex system with a myriad of incentives, disincentives, open and shut doors and windows, gatekeepers, etc. Influencing these structures wields far-reaching intended and unintended consequences. Again, concepts of power inform each other, and the clear and conscious application elucidates practices of science diplomacy. The 1 ^st^ and 2 ^nd^ faces of power above can often contribute to shaping structures of science with wide-ranging intended and unintended consequences. Languages, experiences, traditions, perspectives in research can be promoted or marginalized.

Foucault contributed significantly to theorizing and understanding the relationship between power and knowledge ( Foucault, 1975; Foucault, 1980). Foucault in his archaeology of knowledge concerning punishment, sexuality, mental health, and other areas of society, showed how power shapes what is considered knowledge and truth. Knowledge is a source of power, which then contributes to shaping what is considered truth and knowledge. For science diplomacy as the foreign policy application of science and science collaboration, this interaction between power and knowledge is important. Again, concepts on power overlap and elucidate each other. Power to shape what is considered truth and knowledge between and within societies is an important strategic instrument. Knowledge as a source of power also becomes a foreign policy instrument between countries and societies, where the materially more powerful may be the less knowledgeable and have their power curtailed. The defeat of imperialism is perhaps an illustration of inferior knowledge about the colonized.

Bourdieu contributed significantly to understanding different kinds of capital, social, cultural/education and financial, and how the educational system reproduces such capital, preserving and promoting upper class and upper middle-class groups while excluding working class groups ( Bourdieu, 1979; Bourdieu, 1989). The apparent meritocracy of such processes was termed symbolic violence. International education and research are also marked by social, cultural/educational and financial capital.

Western elite education, especially Anglo-Saxon, accrues the greatest social and cultural/educational capital, while being exclusive and prohibitive in the cost of financial capital. Educational and science diplomacy in terms of giving foreign nationals access to such education and research bestows or denies these different capitals. Colonial powers did so deliberately, and the US has equally used access to US education and research as a foreign policy instrument during and since the Cold War.

InsSciDE brings together political science and Science & Technology Studies (STS), where Sheila Jasanoff’s work on co-production contributes to understanding science diplomacy ( Jasanoff, 2004). STS sheds a clear light on perceptions of science as neutral or objective. Jasanoff’s co-production concept illuminates how social and natural order is co-produced in complex ways, where one should be cautious of unidirectional or monocausal explanations. As such, STS problematizes the science in science diplomacy, as International Relations critically discusses the diplomacy part for any overly optimistic or normative beliefs on diplomacy. The co-production concept in STS contributes to understanding how also in international politics science and diplomacy are co-produced and co-constitutive. Science and technology contribute to ordering international politics, security, diplomacy, etc. Diplomatic practices in turn contribute to ordering science and technology.

The February 2019 review of InsSciDE raised the question of feminist theory and science diplomacy. A literature search of “feminist theory science diplomacy” does not reveal any results. As mentioned by Aggestam and Towns, diplomacy is historically and still a highly gendered activity and has a great overrepresentation of male ambassadors and diplomats in general ( Aggestam & Towns, 2019). Aggestam and Towns propose a research agenda on gender in reconstitution of diplomacy, where they do not mention science diplomacy. This research agenda is valuable for considering gender aspects to science diplomacy.

“Science IN diplomacy” may well have conscious or unconscious gender biases in the knowledge, agendas, priorities, etc., promoted, or marginalized. States and non-state actors may likely pursue gender biased agendas in “diplomacy FOR science.” The transnational epistemic communities of “science FOR diplomacy” are likely to be skewed in terms of gender representation. Such gender biases in different dimensions of science diplomacy practice should be the topic of analysis, theorizing, and policy.

## Science diplomacy and political psychology

A less explored dimension of science diplomacy is the interface with political psychology, which is the application of psychology to understand political processes ( Huddy *et al.*, 2013). Science diplomacy has not been connected with the longstanding research program on political psychology in international politics ( Levy, 2013). This paper will introduce the relation between science diplomacy and key political psychology concepts concerning international politics.

### Science diplomacy, perception and misperception in international politics

Robert Jervis defined much of the political psychology research agenda in international politics in his classic 1976 book, Perception and Misperception in International Politics ( Jervis, 1976). Jervis discusses two possible disastrous misperceptions in international politics: overreacting to an enemy with the outbreak of World War I as example, or not reacting sufficiently to an enemy, where pre-World War II appeasement of Nazi-Germany is the example forming an overly influential analogy.

Broadly speaking, science diplomacy in its main practices should be expected to mitigate these dangers. More science diplomacy in all three practices discussed here should familiarize states and their foreign policy-makers with their counterparts making for more accurate perception and judgment. Especially “science FOR diplomacy” with dense transnational epistemic communities between potential enemies should make for improved decision-making. However, key political psychology concepts caution against unfounded optimism.

### Science diplomacy, learning and socialization

The standard neo-classical assumption in social science is rational decision-makers with Bayesian updating of perceptions in view of new information contradicting previous beliefs. However, research shows this assumption to be unfounded, and individuals’ perceptions are disproportionately shaped by previous learning and socialization ( Levy, 2013). Science diplomacy can affect such previous learning and socialization and therefore later perception and judgment.

There is a long foreign policy tradition of using education of other societies’ elites as a strategic tool. Education of foreign decision-makers can be a way to influence their worldviews, or it can simply be a way to build access and networks. Missionary education illustrates this approach, and state support shows how it has been and remains a foreign policy instrument. Most recently the soft power thinking post-9/11 in War on Terror and US support for American-style higher education in the Middle East reflected such thinking.

“Science FOR diplomacy” with transnational epistemic communities socializes and shapes participants’ views. These views may well be unruly and frustrating for national foreign policy systems and funders; for instance, members of US Congress have been frustrated about overt anti-US and anti-Israel views among students at American universities in the Middle East receiving US federal support ( Newsweek, 1970). However, the ability to socialize and shape and direct worldviews is likely to be a long-term function of material and intellectual resources, where the US and the West in general is at significant advance.

A homogenous group socialized into a certain perspective is likely to be biased and overly shaped by previous experiences, which points to the foreign-policy decision-making pitfall of groupthink.

### Science diplomacy to counter groupthink

Another social psychology term of relevance for science diplomacy is groupthink, the tendency of poor analysis in too homogenous groups because of lacking diversity of perspectives and dissenting views with potential disastrous foreign policy outcomes ( Janis, 1972). “Science FOR diplomacy” can probably mitigate groupthink by bringing together academics from divergent backgrounds and views. Intellectual boycotts and sanctions of a political opponent, as is seen currently concerning Russia over its invasion of Ukraine, risks increasing groupthink on both sides with poorer analysis and decision-making. The US 2001 invasion of Afghanistan or 2003 invasion of Iraq, both failing to achieve their objectives, illustrate the perils of discouraging dissent and basing policy on invalid analysis and assumptions.

### Science diplomacy and time horizons

Time horizons and discounting of the future are important research topics in political psychology and international politics ( Levy, 2013). A rough saying is how democratically elected politicians operate on suboptimal short time horizons, often in comparison with bureaucratic autocracies as the Chinese Communist Party state.

Science is thought to have a longer time horizon than democratic politics. Science diplomacy practices may affect concerns of time horizons in diplomacy and international politics. “Science IN diplomacy” may extend the usual time horizons of politics by long timelines looking back in time and predicting the future. Climate science timelines going thousands of years back and modeling decades into the future is different to the time horizons of especially electoral politics.

“Science FOR diplomacy” also affects time horizons. Deep transnational epistemic communities often rest on decadal scientific cooperation or earlier studies. The deep personal relationships necessary to bridge sharp political crises require trust built over a long time and much interaction. Robert Axelrod’s pioneering game theoretical findings on repeated games as a basis for cooperation and overcoming incentives for defection are relevant here ( Axelrod, 1990). Repeated games as a basis for long-term collaboration is relevant to understanding interpersonal “science FOR diplomacy”. Such repeated games may also contribute to explain inter-state outcomes in “diplomacy FOR science,” how states build trust to engage in scientific cooperation.

## Science diplomacy and world order

Academic International Relations commonly looks at international politics in terms of the structure of the international system of states, the nature of that system, and the distribution of relative power between the most powerful states. The nature of this system is anarchic, which means that there is no world government to impose law and order and protect states from each other. International law is circumscribed by great power interests, and superpowers and great powers will transgress international law, when they deem it to be in their interest, as illustrated by the US invasion of Iraq in 2003 or the Russian 2022 invasion of Ukraine.

The relative distribution of power between great powers greatly influences international politics. An international system can be multipolar with three or more great powers as was roughly the case before World War I. With two overwhelming superpowers, the system is bipolar as with US-Soviet competition during the Cold War. The system was unipolar after the Cold War and the dissolution of the USSR with the US as the single superpower. US unipolarity and hegemony (the US could and did formulate rules and regimes) was the basis of globalization creating global market, science, and technology integration.

Globalization in turn undermines US unipolarity, because globalization furthered a historical normalization. Asia in general and China represent very large parts of global economic output. Russia returns from the depths of its 1990s and early 2000s socio economic crisis as the Eurasian great power it has been since Peter the Great. The post-Cold War US unipolar world order is therefore under great strain as evident in Sino-American competition and now the war on Ukraine.

Science and technology have, especially since the 1900s, been a key aspect of great power competition. With WWI as the first total, industrial world war, science and technology capacity became major determinants of war outcomes. The role of science and technology increased with WWII, which saw the development of technologies such as nuclear weapons, ballistic and cruise missiles, and radar. These technologies played key roles in the bipolar US-Soviet Cold War competition.

The Cold War was a science and technology competition besides ideological, economic, military, etc., where the two superpowers competed fiercely in nuclear and space domains among others. They competed for scientific and technological prestige. They used science and technology to order and discipline their respective bloc. Science and technology exchange with the other bloc was strictly disciplined, but also used for intelligence purposes. Cold War science diplomacy was both inside the two competing blocs, between them, and competing for non-aligned countries. Science diplomacy between the two blocs was most notably in arms control, as “science IN diplomacy” with the extremely technical nature of arms control, and “science FOR diplomacy” with transnational communities such as the Pugwash conferences.

Post-Cold War US unipolarity and hegemony with the absence of superpower competition made way for globalization, also of science and technology with extensive cooperation and exchange, also between the West and China and Russia. This exchange contributed to the historical normalization of especially China’s relative position in the world economy and Russia’s return as a Eurasian great power, which undercut US unipolarity and hegemony.

Post-Cold War US unipolarity and hegemony made it possible in the science and technology domain to focus on global challenges, where climate change is the clearest and pressing. The US was also the sole superpower of science, technology, and higher education with its predominant science, technology, and higher education sector, and English as the central language of the global language system (Swaan). At the same time, domestic political and social crises in American society have limited the US’ ability to ratify the Kyoto Protocol, led to the election of President Donald Trump and withdrawing the US from the Paris Accord, and strong anti-science currents in American society. The tensions between the size and quality of American science, technology, and higher education, and political and social crises in American society reflect American politics.

Globalization with the rise of emerging markets, most notably the BRICS (Brazil, Russia, India, China, South Africa), also undermined US unipolarity in science and technology. The West is a small minority of the world population and by far the largest parts of human ingenuity and talent lie outside the West. As social and political conditions allow larger parts of humanity to express its ingenuity and talent, the relative importance of the US and the West shrinks, also in science and technology.

Even post-Cold War US unipolarity and hegemony did not allow us to address the most pressing global challenge of climate change, which should have been easier with one superpower at the top of the table. This inability was also, to a significant extent, a product of domestic American crises. The emerging world order with much intensified competition between especially the US and China, but also between the US and Russia, which has developed into large-scale proxy war in Ukraine, will make addressing global challenges such as climate change much more difficult. Other global challenges requiring all dimensions of science diplomacy are, for instance, arms control, pandemics, cyberspace, and space governance.

## Science diplomacy for emerging Sino-American loose bipolarity

China and the US are by far the world’s two largest national economies, so the international system is starting to express bipolar traits ( Tunsjø, 2018). Compared to the early Cold War with a destroyed Europe and Japan, the emerging Sino-American bipolarity is “looser” with stronger secondary actors such as the EU, Groups of Seven or 20, or BRICS. Sino-American bipolarity or a more multipolar world is more dangerous in the international security domain than US unipolarity, which is clear from the Ukraine war. The ultimate catastrophe would be a Sino-American war over the status of Taiwan. Addressing global challenges is harder in general under bipolarity or multipolarity, and especially with actual or overhanging risk of great power (proxy) war.

Science diplomacy practices can contribute to mitigate global challenges under Sino-American bipolarity. “Science IN diplomacy” is necessary to address very complex global challenges of climate change, multipolar arms control, pandemics, cyberspace, and space governance. Competing superpowers must overcome their animosity to allow “diplomacy FOR science” to facilitate both “science IN diplomacy” and “science FOR diplomacy.” Transnational epistemic communities of “science FOR diplomacy” are probably necessary to mitigate these global challenges between opposing superpowers and their blocs with very different cultural, political, and social systems. The cultural, political, and social differences today between the US (and the West) and China (and BRICS countries in general) is probably greater than between the US and the USSR adding uncertainty and complexity.

As reiterated about state power and science diplomacy, states need to support and tolerate science diplomacy with the enemy for science diplomacy to work. Closed societies such as China and Russia increasingly curtail intellectual relations with the West, probably for domestic political stability reasons. The US and the West won the Cold War because their open societies provided their citizens with better and more attractive living conditions than the Soviet alternative. The West must keep this lesson in mind, fighting instincts to respond to closedness with closedness and keeping ideas and knowledge hidden from the enemy. The West should keep in mind that openness even in face of closedness is a long-term strategic asset.

## Science diplomacy practices under different world orders

The world has not known multipolarity since before WWI or WWII, and it has never known a multipolar system with nuclear weapons, which would theoretically be unstable and offer significant arms control challenges. Pre-WWI/II multipolarity was overwhelmingly dominated by Western colonial empires, but these had an integrated and vibrant scientific system, a “republic of letters,” which was nonetheless incapable of averting the catastrophes of WWI and WWII.

A future true multipolarity with two or more peer-competitors to the US, which could be a truly federal Europe or India on par with China, would entail sharp science and technology competition for structural reasons. The Sino-American science and technology competition has already been clear in as mundane a field as mobile phones, where a major and popular Chinese manufacturer, Huawei, has been pushed out of Western markets.

Science diplomacy under nuclear multipolarity would be necessary to manage global challenges as under the emerging Sino-American loose bipolarity and would face the same challenges of temptations of closedness towards competing great powers and their blocs.

## Science diplomacy as the dependent variable

Science diplomacy can also be the dependent variable to be explained rather than the independent variable explaining diplomatic outcomes. Science diplomacy as a dependent variable then lends insight into why “science IN diplomacy”, “diplomacy FOR science”, or “science FOR diplomacy” is carried out.

The independent variable explaining science diplomacy as a dependent variable becomes foreign policy decision-makers and processes. What and who explains how science is used IN a certain diplomatic process, for instance the major United Nations environmental conventions on biodiversity or climate change or arms control?

“Diplomacy FOR science” becomes a question of explaining why states engaged in negotiations to facilitate and further scientific cooperation. One example could be the 2017 Agreement on Enhancing International Arctic Scientific Cooperation negotiated between the eight member states of the Arctic Council.

Why do states support and tolerate transnational scientific relations in “science FOR diplomacy”? It is clear that states often do not tolerate such relations, as now illustrated in the academic boycott of Russia over the Ukraine war. For instance, Norway has supported academic cooperation with Russia (and previously the USSR) relatively generously, until now. This Norwegian policy was the reassurance counterpart to the deterrence leg in Norway’s policy towards the USSR and now Russia.

Looking at science diplomacy as the dependent variable to be explained generally points to science diplomacy as a foreign policy tool to achieve foreign policy goals. Science diplomacy becomes an intermediary variable between foreign policy actors and their ultimate goal. In this way science diplomacy as an independent or dependent variable comes together.

## Future directions for theorizing science diplomacy

Science diplomacy is a contested concept, perhaps especially because science is a particularly contested concept. Science diplomacy empirical practice is historically and currently very rich. Both characteristics of science diplomacy complicate theorizing it.

The way forward to theorize science diplomacy is probably through an ideal-type view of science diplomacy practices, which creates order in the empirical richness, and linking these practices with diplomacy analytically, and not normatively. A clear view of science diplomacy practices as independent, intermediary, or dependent variable will also provide a basis for theorizing science diplomacy.

Linking science diplomacy practices to diplomatic outcomes can provide the basis for attempting to theorize any causal relationship. Likewise, an explanation for science diplomacy practices themselves can be sought.

H2020 research on science diplomacy in the EL-CSID, S4D4C and InsSciDE ( Mays *et al.*, 2022) consortia have provided rich historical material and ongoing discussions for continued work to define and theorize science diplomacy.

## Ethics and consent

Ethical approval and consent were not required.

## Data Availability

No data are associated with this article.

## References

[ref-1] Adler E Haas PM : Conclusion: epistemic communities, world order, and the creation of a reflective research program. *International Organization.* Knowledge, Power, and International Policy Coordination (Winter),1992;46(1):367–390. 10.1017/S0020818300001533

[ref-2] Aggestam K Towns AE : The gender turn in diplomacy: a new research agenda. *Int Fem J Polit.* 2019;21(1):9–28. 10.1080/14616742.2018.1483206

[ref-3] Axelrod RM : The Evolution of Cooperation.Penguin Books, London; New York.1990. Reference Source

[ref-4] Bachrach P Baratz MS : Decisions and nondecisions: an analytical framework. *Am Polit Sci Rev.* 1963;57(3):632–642. 10.2307/1952568

[ref-5] Bachrach P Baratz MS : Two faces of power. *Am Polit Sci Rev.* 1962;56(4):947–952. 10.2307/1952796

[ref-6] Baldwin DA : Power and international relations.In: *Handbook of international relations.* eds. W. Carlsnaes, T. Risse & B.A. Simmons, Sage Publications, London,2002;77–191. 10.4135/9781446247587.n11

[ref-7] Bertelsen RG : American missionary universities in China and the Middle East and American philanthropy: Interacting soft power of transnational actors. *Global Society.* (Special Issue: American Philanthropy and the Hard, Smart and Soft Power of the United States):2014a;28(1):113–127. 10.1080/13600826.2013.848188

[ref-8] Bertelsen RG : The university as a transnational actor with transnational power: American missionary universities in the Middle East and China. *PS: Political Science & Politics.* 2014b;47(3):624–627. 10.1017/S1049096514000754

[ref-9] Bertelsen RG : Private foreign-affiliated universities, the state and soft power: the American University of Beirut and the American University in Cairo. *Foreign Policy Analysis.* 2012;8(3):293–311. 10.1111/j.1743-8594.2011.00163.x

[ref-10] Bourdieu P : La noblesse d’État. Grandes écoles et esprit de corps. Editions de minuit, Paris,1989;568. 10.7202/1002099AR

[ref-11] Bourdieu P : La distinction: critique sociale du jugement.Éditions de minuit, Paris.1979. Reference Source

[ref-12] Dahl R : Who Governs? Democracy and Power in an American City.Yale University Press, New Haven, CT.1961. Reference Source

[ref-13] Danish Security and Intelligence Service (PET): Is your research at risk?.[Homepage of Danish Security and Intelligence Service (PET)].2021; 2021-05-last update. Reference Source

[ref-14] Eddé C : L'USJ: portrait d'une université.Presses de l'Université Saint-Joseph, Beyrouth.2000.

[ref-15] Fägersten B : D2.6 European Science Diplomacy Strategy.H2020 Inventing a shared Science Diplomacy for Europe, Paris.2021.

[ref-16] Fairbank JK : The Missionary Enterprise in China and America. Harvard University Press, Cambridge, Massachusetts.1974. Reference Source

[ref-17] Foucault M : Power/knowledge: selected interviews and other writings, 1972-1977.Pantheon Books, New York.1980. Reference Source

[ref-18] Foucault M : Surveiller et punir. Naissance de la prison,Gallimard, coll. Bibliothèque des histoires, Paris.1975. Reference Source

[ref-19] Gluckman PD Turekian VC Grimes RW : Science Diplomacy: A Pragmatic Perspective from the Inside. *Sci Dipl.* 2017;6(4). Reference Source

[ref-41] Guzzini S : Structural power: The limits of neorealist power analysis. *International Organization.* 1993;47(3):443–478. 10.1017/S0020818300028022

[ref-20] Haas PM : Introduction: epistemic communities and international policy coordination. *International Organization.* 1992;46(1):1–35. 10.1017/S0020818300001442

[ref-21] Huddy L Sears DO Levy JS (eds) : The Oxford Handbook of Political Psychology.2nd edn, Oxford University Press, Oxford,2013. 10.1093/oxfordhb/9780199760107.001.0001

[ref-22] Janis I : Victims of Groupthink: A Psychological Study of Foreign Policy Decisions and Fiascoes.Houghton Mifflin, Boston,1972. Reference Source

[ref-23] Jasanoff S (ed) : States of Knowledge: The Co-production of Science and the Social Order.Routledge,2004. Reference Source

[ref-24] Jervis R : Perception and misperception in international politics.Princeton University Press, Princeton, N.J,1976. 10.2307/j.ctvc77bx3

[ref-25] Langenhove LV : Tools for an EU Science Diplomacy.Directorate-General for Research and Innovation (European Commission), Brussels,2017. Reference Source

[ref-26] Levy JS : Psychology and Foreign Policy Decision-Making.In: *The Oxford Handbook of Political Psychology.* 2nd edn, Oxford University Press, Oxford, pp. Huddy, Leonie; Sears, David O.;Levy, Jack S.;.2013. 10.1093/oxfordhb/9780199760107.013.0010

[ref-27] Lord C : Losing hearts and minds? public diplomacy and strategic influence in the age of terror.Praeger Security International, Westport, CT,2006. Reference Source

[ref-28] Lukes S : Power: a radical view.The Macmillan Press Ltd, London,1974. Reference Source

[ref-29] Mays C : Inventing a shared science diplomacy for Europe: Twenty-eight historical cases, a thousand ideas.In: *Inventing a shared science diplomacy for Europe: Interdisciplinary case studies to think with history.* eds. C. Mays, L. Laborie & P. Griset, *Zenodo.* 2022. 10.5281/zenodo.6639894

[ref-30] Mays C Laborie L Griset P (eds) : Inventing a shared science diplomacy for Europe: Interdisciplinary case studies to think with history. *Zenodo.* 2022. 10.5281/zenodo.6590097

[ref-31] Newsweek: Guerrilla U.Newsweek, New York,1970.

[ref-32] Nye JS Jr : The future of power.PublicAffairs, New York,2011. Reference Source

[ref-33] Nye JS Jr : Soft power: the means to success in world politics.1st ed edn, Public Affairs, New York,2004. Reference Source

[ref-34] Parmar I : Foundations of the American Century: The Ford, Carnegie and Rockefeller Foundations and the rise of American power.Columbia University Press, New York, NY,2012. Reference Source

[ref-35] Rugh WA : American encounters with Arabs: the "soft power" of U.S. public diplomacy in the Middle East.Praeger International Security, Westport, CT,2006. Reference Source

[ref-36] The Royal Society & AAAS: New frontiers in science diplomacy: Navigating the changing balance of power.The Royal Society; AAAS, London; Washington, DC,2010. Reference Source

[ref-37] Tunsjø Ø : The Return of Bipolarity in World Politics: China, the United States, and Geostructural Realism.Columbia University Press, La Vergne,2018. Reference Source

[ref-38] Weber M : Wirtschaft und Gesellschaft.Mohr, Tübingen,1921-1922.

[ref-39] West P : Yenching University and Sino-Western Relations, 1916-1952.Harvard University Press, Cambridge, MA,1976. Reference Source

[ref-40] Xu EY : Liberal arts education in English and campus culture at St. John's University.In: *China's christian colleges: cross-cultural connections, 1900-1950.* eds. D.H. Bays & E. Widmer, Stanford University Press, Stanford, CA,2009;107–124.

