# Supplementary material for: Social theory and science diplomacy
Source: Open Res Eur. 2024 Jan 8;4:10. [Version 1] doi: 10.12688/openreseurope.15020.1 (PMC10882199; doi:10.12688/openreseurope.15020.1)
Supplement: Peer review history

# Reviewer report: Reviewer response for version 1

**DOI:** 10.21956/openreseurope.16239.r37435

**Published:** 2024-02-21

**Authors:** Pierre-Bruno Ruffini

**Affiliations:** 1 Faculty of International Affairs and EDEHN (Equipe D’Economie—Le Havre Normandie), University of Le Havre Normandie, Le Havre, France

Professor Rasmus Bertelsen (RB), from UiT-Arctic University of Norway, has accustomed us to expressing ideas and developing analyzes on the subject of science diplomacy (SD) that challenge simplistic representations, and this is what this subject really needs. His essay “Social theory and science diplomacy" does not escape this rule. RB’s input is essentially conceptual (how to define and theorize SD), considering that “it is not feasible to develop one theory of science diplomacy”. He rightly recognizes that many disciplines must be mobilized to understand and analyze SD. Here are my comments on this very inspiring essay.

 I - The overall framework in which the author situates his reflections can be described in four points:

1. The essay ranks among the critical views of the dominant narrative of SD. By openly recalling the political nature of science as a social practice, the author calls into question this narrative which overplays the reference to the supposed neutrality of science to affirm the pacifying virtues of international scientific cooperation.
2. The author notes that the word “diplomacy” governs the expression “science diplomacy”. This would deserve a longer development. But I can only agree with an idea which is familiar to me. I share with the author the view that SD is first and foremost diplomacy.#
3. By favoring a vision of SD “as pursuit of state foreign policy interest”, the author takes inspiration from the realist school of international relations (he refers in the text to the "anarchic nature of international politics”, to the “anarchic nature of the international system”). He considers that states are the dominant although not exclusive actors in international relations.
4. The theory of power plays an important place in his analysis of SD. The power in question (power over, power with) is that of states on the international scene.

II - The essay is made up of a succession of themes which are not explicitly linked to each other, but about which RB brings added value by suggesting an original reading or by asking a new question. His text is a reservoir of ideas, which is very useful for building a research agenda on SD.

 By moving from one theme to another, RB point out several “blind spots” that theoretical reflections on SD have so far left aside, among which Jasanoff’s co-production concept in STS, which could contribute to understand SD practices; the "gender biases in different dimensions of science diplomacy practice"; actors’ interests in the agenda setting of science *in* diplomacy.

 On other themes, the contribution is more substantial. Some of these particularly caught my interest and led to comments that follow.

**1. Definitions:**

 The author starts with defining SD as “the use of science for foreign policy purposes”. Some would find this definition too restrictive, as it limits SD to “science for diplomacy” (science as a means, and foreign policy as an objective).

 The author states that “Science FOR diplomacy” corresponds to the transnational epistemic communities between societies created through scientific activities”. This view surprised me. The organization of science in transnational epistemic communities can indeed facilitate the exercise of “S for D”. But can a definition of S for D fail to refer to state interests and foreign policy, all the more so when having in mind the author’s definition of SD? Another surprising statement (in the table) is that “scientists do not usually represent their state, protect state interests, nor formally negotiate for their state, which would bring these scientists outside “science FOR diplomacy.” This calls into question the importance of transnational epistemic communities in “S for D”. However, the author also notes that “Epistemic communities are noteworthy for their agenda-setting power on a policy question”. These contrasting remarks make it necessary to specify the definition of S for D in this essay.

**2. Independent and dependent variables:**

 RB makes a wide use of the 2010 three-pole definition of SD and shows that new perspectives can be drawn from it. He reflects on the status of independent (explanatory) variable versus dependent (explained) variable of D (diplomacy) and of S (science) in the interrelations between S and D. He then looks at SD as an independent, intermediate, or dependent variable, which is a newer question.

 The author states that “S in D” can be seen as an independent variable explaining the dependent variable “foreign policy outcome”. Conversely, he states that “S in D” can be seen as a dependent variable. But what then is the explanatory variable?

 The author does not discuss the possibility of a dual status (independent/dependent) of the variable “S for D”, as one might have expected.

 Finally, he labels “D for S” as an “intermediate variable” in the sense that “D for S” would explain “S in D” as well as “S for D”. Unless one considers that this is just a slightly complicated way of saying that there is some D involved in "S in D" and "S for D", it would be good to further develop this idea of " intermediate variable” (all the more that the reference made here to Fägersten 2021 is not illuminating).

**3. The independence of non-state actors:**

 The author writes about “The *apparent* independence of non-state actors in science diplomacy”, and indicates that “Non-state actors (…) can engage in science diplomacy *seemingly* independent of the state” (emphasis is mine). These formulations suggest that independence of non-state actors is only an illusion. This could be discussed.

 SD initiatives ultimately fall indeed under the “pursuit of state foreign policy interest”, as RB writes, but this does not imply that non-state actors who participate in such initiatives lose their independence. Generally speaking, nothing obliges a university or a research center - even if they are financed by public money - to enter into an international cooperation program which has political-diplomatic objectives in addition to its academic and scientific objectives (e.g. the participation in the 1990s of Western European universities in Tempus programs with Eastern European universities – these programs being part of the political approach of bringing Eastern European countries into the sphere of European influence). A higher education or research institution can be associated – some would say “instrumentalized” – to a “top down” SD initiative without losing its independence. The author is right however on one point: “… in times of deep crisis…” it could be different and the last word goes to foreign policy interests (see the example of academic sanctions against Russia, cited several times in the text).

**4. Political psychology:**

 Another added value of the essay is to introduce political psychology for understanding SD. After recalling one of the arguments of the psychological approach to international relations (misperceptions leading tooverreacting to an enemy or not reacting sufficiently to an enemy) RB writes: “science diplomacy in its main practices should be expected to mitigate these dangers”. The author recalls the expectation expressed in the SD dominant discourse: because they are transnational by nature, epistemic communities can alleviate tensions between potential enemies. The author mentions the concept of “co-production” in his text, and good use could be made of it here. How does the psychology of actors intervene in the co-production (or co-construction) of practices by scientists and diplomats? A path is opened in the text when reminding that the time horizons of scientists and political-diplomatic actors are not the same. The author writes that “Science IN diplomacy” can extend the usual time horizons of politics by long timelines looking back in time and predicting the future”. Likewise, the author mentions “Repeated games as a basis for long-term collaboration”, and states that “The deep personal relationships necessary to bridge sharp political crises require trust built over a long time and much interaction”. One can admit that, but how to go further? Can we establish that through interacting with science issues and scientists in international conferences and in the governance of global issues, diplomats could adjust their own time scale? Can we establish that the psychology of scientific actors (driven by the “values” of science) can promote the building of trust and exert a positive influence on international political relations? The author does not answer these questions, but his remarks are helpful for pointing out another blind spot in the analysis of SD.

 Furthermore, the author states that SD can counter groupthink. He states that “Science FOR diplomacy can probably mitigate groupthink by bringing together academics from divergent backgrounds and views.” But we could turn the argument around here, and argue that the dominant narrative on SD was built according to a logic of groupthink, within the sphere of practitioners who wrote on SD and promoted a militant and exaggeratedly optimistic vision of the SD.

**5. Science diplomacy and world order - The US-China relationship:**

 The US-China relationship is discussed in the last section of the essay. After noting the “actual or overhanging risk of great power (proxy) war”, the author writes that “science diplomacy practices can contribute to mitigating global challenges under Sino-American bipolarity”. But can we go further than an affirmation of principle? Once again, the potential of transnational epistemic communities is mentioned by the author. We should here differentiate between two things: retaining scientific cooperation links between rival countries, and the capacity of this to reduce tensions between rival countries. We have historical (Cold War) and contemporary examples showing that scientific cooperation can continue between countries in situations of political and ideological confrontation. But the capacity of international scientific cooperation to alleviate political tensions remains a hypothesis to be verified, case by case. Ultimately, it is always the same nagging question that is asked: that of the pacifying virtues of international scientific cooperation.

Is the argument persuasive and supported by appropriate evidence?

Partly

Is the work clearly and cogently presented?

Yes

Is the topic of the essay discussed accurately in the context of the current literature?

Yes

Does the essay contribute to the cultural, historical, social understanding of the field?

Yes

Reviewer Expertise:

Science diplomacy

I confirm that I have read this submission and believe that I have an appropriate level of expertise to confirm that it is of an acceptable scientific standard.

# Reviewer report: Reviewer response for version 1

**DOI:** 10.21956/openreseurope.16239.r37436

**Published:** 2024-02-16

**Authors:** Kleinsy Bonilla

**Affiliations:** 1 OWSD LAC Institution: Organization for Women in Science for the Developing World (Ringgold ID: 460943), State University of Campinas, Trieste, Friuli-Venezia Giulia, Italy

The essay is clear, well written and represents a good contribution to the endless debate about definitions and conceptualizations of Science Diplomacy. Indeed, as the author points out, literature addressing practice on Science Diplomacy is abundant, an so is the relevance on discussing the challenges of theorizing. The author accurately suggest that social theory  brings order to rich empirical material and links science diplomacy practices to diplomacy analytically rather than normatively. Even more so, when conceptualizing and defining concepts and categories in Science Diplomacy is highly context-relevant. For example, understanding on Science Diplomacy differs in the Global South and North. In summary, I recognize value and contribution in the topic, which is presented timely with an easy to follow structure.

Is the argument persuasive and supported by appropriate evidence?

Yes

Is the work clearly and cogently presented?

Yes

Is the topic of the essay discussed accurately in the context of the current literature?

Yes

Does the essay contribute to the cultural, historical, social understanding of the field?

Yes

Reviewer Expertise:

science and technology policies; Science Diplomacy in the Global South; Women in Science

I confirm that I have read this submission and believe that I have an appropriate level of expertise to confirm that it is of an acceptable scientific standard.
